# Low-Temperature and UV Irradiation Effect on Transformation of Zirconia -MPS nBBs-Based Gels into Hybrid Transparent Dielectric Thin Films

**DOI:** 10.3390/gels8020068

**Published:** 2022-01-20

**Authors:** Viorica Muşat, Elena Emanuela Herbei, Elena Maria Anghel, Michael P. M. Jank, Susanne Oertel, Daniel Timpu, Laurenţiu Frangu

**Affiliations:** 1 Centre of Nanostructures and Functional Materials, Faculty of Engineering, Department of Materials and Environmental Engineering, University “Dunărea de Jos” of Galaţi, Domneasca 111, 800201 Galaţi, Romania; elena.herbei@ugal.ro; 2 Institute of Physical Chemistry, “Ilie Murgulescu” of Romanian Academy, Spl. Independenţei 202, 060021 Bucharest, Romania; 3 Fraunhofer Institute for Integrated Systems and Device Technology IISB, Schottkystraße 10, 91058 Erlangen, Germany; Michael.Jank@iisb.fraunhofer.de (M.P.M.J.); susanne.oertel@iisb.fraunhofer.de (S.O.); 4 Photochemistry and Polyaddition Department, “Petru Poni” Institute of Macromolecular Chemistry, 700487 Iaşi, Romania; dtimpu@icmpp.ro; 5 Faculty of Automation, Computers, Electrical Engineering and Electronics, “Dunarea de Jos” University of Galati, Ştiinţei 2, 800210 Galaţi, Romania; Laurentiu.Frangu@ugal.ro

**Keywords:** modified sol-gel, nBB-based hybrid, hybrid gel film, zirconia, PMMA, high-k dielectric thin film, transparent flexible electronics

## Abstract

Bottom-up approaches in solutions enable the low-temperature preparation of hybrid thin films suitable for printable transparent and flexible electronic devices. We report the obtainment of new transparent PMMA/ZrO_2_ nanostructured -building blocks (nBBs) hybrid thin films (61–75 nm) by a modified sol-gel method using zirconium ethoxide, Zr(OEt)_4_, and 3-methacryloxypropyl trimethoxysilane (MPS) as a coupling agent and methylmethacrylate monomer (MMA). The effect of low-temperature and UV irradiation on the nBBs gel films is discussed. The thermal behaviors of the hybrid sols and as-deposed gel films were investigated by modulated thermogravimetric (mTG) and differential scanning calorimetry (DSC) analysis. The chemical structure of the resulted films was elucidated by X-ray photoelectron (XPS), infrared (IR) and Raman spectroscopies. Their morphology and crystalline structure were observed by scanning electron microscopy (SEM), high-resolution transmission electron microscopy (HR-TEM), and grazing incidence X-ray diffraction. The cured films show zirconia nanocrystallites of 2–4 nm in the hybrid matrix and different self-assembled structures for 160 °C or UV treatment; excellent dielectric behavior, with dielectric constant values within 6.7–17.9, depending on the Zr(OEt)_4_:MMA molar ratio, were obtained.

## 1. Introduction

Thin-film transistors (TFTs) are the key component of the flat-panel displays and have been traditionally realized with amorphous silicon (a-Si) semiconductors. Research into new metal oxide-based semiconductors has recorded a major upsurge in quest for increased performance and functionality [1,2]. Higher resolution, large electron mobility, good transparency, and long-lasting stability are reported for the new materials in contrast with Si [2]. Paving the way towards printability, solution processible metal oxides have to be integrated with complementary interfacing materials to deliver optimized contact performance [3] and channel control [1].

For the latter, hybrid materials combining properties of the organic polymers (flexibility, elasticity, adhesion to substrate, facile processing, low leakage) with those of the inorganic compounds (high permittivity, optical and thermal properties) can simultaneously meet all the requirements of the gate dielectric for use in transparent and/or flexible electronics [1,2,3,4]. Hybrid insulators with high dielectric constant, “high-k”, directly foster the development of TFTs and circuits with improved high-frequency behavior and area efficiency. When both organic and inorganic components have dielectric properties, value of the bandwidth energy and consequently the dielectric properties of a hybrid material can be tailored by controlling ratio and bonds between components. According to the type of organic-inorganic interactions, hybrid materials are type I and II, with weak (Van der Waals, hydrogen) and strong (covalent and ionic) interactions, respectively [4]. Hybrid materials may consist of alternating layers of organic compound and inorganic compound (multilayer hybrid structure) or the two components (organic and inorganic ones) covalently linked to each other, usually by means of a coupling agent [5], and dispersed at nanometer or molecular scale within the same layer [1].

Vapor-based (chemical vapor deposition, CVD, including atomic layer deposition, ALD, [6]) and solution-based (self-assembly, hybrid metal–organic frameworks and nano-building block approach, nBBs [7], etc.) chemical routes are the known methods for obtaining multilayer hybrid materials by successive deposition of organic and inorganic layers. High cost is the main drawback of CVD production of the metal oxide transistors [2]. Chemical routes rely on several processes as copolymerization of block copolymers macromolecules, metal alkoxides and organosilans (coupling agents), functionalization of the inorganic nanofillers as well as encapsulation of the organic components within sol-gel derived metal oxides [7,8]. In the case of single-layer hybrid materials, in situ and ex-situ chemical methods are employed [1]. In -situ polymerization enables obtaining of homogenous layer which might contain byproducts in contrast with ex-situ methods when homogeneity of the coating is difficult to achieved [7].

Simplicity and low cost of the solution processed (soft chemistry route) hybrid materials are quite attractive for large-area flexible electronics [2]. Among solution methods, the sol-gel method has the greatest potential due to its low processing temperatures and combination of different types of chemical compounds while homogeneous structures at molecular scale are obtained [8,9,10]. Lately, vacuum and photolithographic techniques for production of high-quality transparent and flexible electronics have been replaced by the solution-base printed technique [11]. The latter one implies adequate reagents selection for sol preparation [12], low processing temperature (<200 °C), and UV curing [13,14,15,16,17] of as-deposed gel films. Thus, different sol-gel functional films, dielectric, semiconductor and conductive ones, etc., for electronic, opto- and/or bioelectronic devices have been targeted by the printing method.

Polymethylmethacrylate polymer (PMMA)-oxide materials are the most known hybrid materials [2,5]. Oxides as Ta_2_O_5_ [18], Y_2_O_3_ [19], Al_2_O_3_ [20], and ZrO_2_ named high-k dielectrics, have been used to obtain hybrid films by sol-gel method [9,21,22,23]. Our early hybrid films using metal oxide precursors were obtained by in situ generation tantalum or zirconium oxide nanocrystallites as inorganic component [24,25]. Due to its outstanding properties (high optical transparency, dielectric constant k of 25 and energy band gap within 5.1–7.8 eV) [2], ZrO_2_ stands of obtaining hybrid materials with numerous applications. Di Maggio et al. and Girardi et al. reported synthesis of zirconium oxo clusters- based inorganic-organic hybrid coatings for wood [13] and aluminum alloys [17] protection.

This work focuses on a new class of hybrid high-k dielectric transparent films based on zirconium oxide in the PMMA polymer matrix. The hybrid materials obtained at low temperature by in situ sol-gel reactions between Zr(OEt)_4_ modified with MPS ligand and MMA were used in preparation of thin films for electronic applications. Two different post-deposition treatments of the hybrid thin films, thermal, and UV exposure, were applied. Properties (optical, thermal, and electrical) along with structural and textural information (derived from IR, Raman, XPS, XRD, SEM, TEM, and AFM techniques) of the hybrid films were assessed and discussed in terms of precursor ratio, type and duration of post-deposition treatment.

## 2. Materials and Methods

### 2.1. Materials

Methylmethacrylate reagent grade 99% (MMA), 3-methacryloxypropyl trimethoxysilane (MPS) reagent grade 98%, and absolute ethanol (Et-OH) 99.99% from Sigma-Aldrich Chemical Company, Inc. (St. Louis, MO, USA.) were used without other processing steps. Zirconium ethoxide, Zr(OEt)_4_, precursor (99.9%) was obtained from Cambridge Multivalent Ltd. (Cambridge, UK).

### 2.2. Sol-Gel Preparation of Hybrid Films

**Figure 1:**
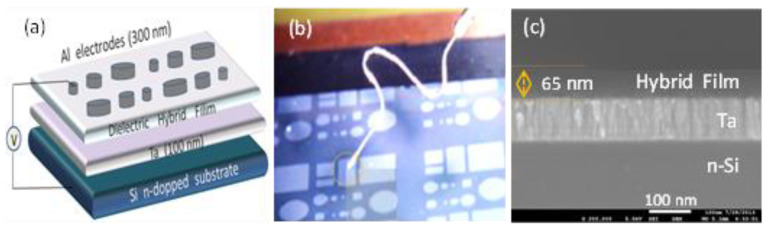
Schematic representation of metal–insulator–metal (MIM) structure (including dielectric hybrid film) used for electrical measuring (**a**), digital imagine (detail of experimental set-up) on the surface of the obtained hybrid film with top Al electrodes (**b**) and SEM cross-section imagine of n-Si/Ta//hybrid film structure (**c**).

Sol preparation started with dissolution of zirconium ethoxide, Zr(OEt)_4_, into absolute ethanol at room temperature. The MPS coupling agent and MMA were added successively according to Zr(OEt)_4_:MPS:MMA molar ratios of 1:1:1 (samples S1) and 6:1:1 (Samples S6), respectively. Each sol was aged under refluxing at 50 °C until it became transparent and then aged for an additional 24 h to deliver the appropriate viscosity for gel film deposition. The aged homogeneous sols were spin-coated (3000 rpm) onto cleaned n-doped silicon substrates covered by sputtering with a tantalum layer of 100 nm thickness (Figure 1). The sputtered tantalum layer was obtained in a mixture of Ar (99.999%) and N_2_ (99.999%) at 9.0 × 10^−4^/Pa background pressure and 200 W DC applied power, using a Ta target of 99.95% purity. The as-deposed gel films were dried for 10 min. at 100 °C onto a hot-plate in air. The final post-deposition treatment of the dried gel films consisted in 10 min. hot-plate heating in air at 160 °C (samples denominated S1-T and S6-T) or 30 min. ultraviolet exposed samples, S1-UV and S6-UV. The UV source with a wavelength of 254 nm emitted by a H 466.1 UV lamp from Herolab GmbH Laborgeräte (Wiesloch, Germany) was located at 5 cm distance far from the samples.

### 2.3. Characterizations

The as-prepared sols, as well as the gel layer resulted by sol heat treated at different conditions, were characterized by modulated thermogravimetric (mTGA) and differential scanning calorimetry (mDSC) analysis. The TGA and DSC curves were recorded in synthetic air (5.0 purity) at 5 K/min. heating rate, using Q 5000IR and Q20, respectively, Thermal Analysis (TA) equipment from TA Instruments, New Castle, DE, USA.

The surface and cross-section morphology of the hybrid films were investigated by scanning electron microscopy (SEM) using a JEOL JSM-7500F/FA microscope from Peabody, MA, JOEL Ltd. USA.

The transmission electron microscopy (TEM) images and data were obtained using a TecnaiTM G2 F30 S-TWIN electron microscope (Hillsboro, OR, USA), equipped with STEM/HAADF detector, EDX (energy dispersive X-ray analysis) and EFTEM—EELS electron loss spectrometer energy loss spectroscopy. Thin film samples were analyzed by TEM using a support table for the five-axis motion of samples. The thin film was supported by a special thin film holder, which was placed on the support table. The sample was then aligned so that the source-sample-detector system was on the same line.

The surface morphology and phase composition of the resulted thin films were also investigated with nanometer scale resolution by atomic force microscopy (AFM) using a NT- MDT NTEGRA Spectra Scanning Probe Microscope (AFM), model SOLVER PRO-M, from NT-MDT, Moscow Russia, in semi-contact approach. Measurements were performed in air at room temperature in clear room.

X-ray diffraction (Thermo Fisher, Waltham, MA, USA) analysis using a PANalytical Empyrean diffractometer at room temperature, with a Cu X-ray tube (λ Cu Kα1 = 1.541874 Å). The measurements were performed using the “Grazing incidence” mode at an angle of incidence ω of 0.5 degrees and angle 2θ within the range of 10–80°.

X-ray photoelectron spectroscopy (XPS)—analysis was used to determine the chemical states of the elements present on the surface and, after quantitative analysis, to find out the element and the chemical state relative concentrations, as well. It is appropriate to note here that all the calculations were performed assuming that the samples were homogeneous within the XPS detected volume (<10 nm). We have to emphasize that the errors in our quantitative analysis (relative concentrations) were estimated in the range of ±10%, while the accuracy for binding energies (BEs) assignments was ±0.2 eV.

Dielectric spectroscopic/Impedance analysis measurements of the films under discussion were carried out by device supplied by Novocontrol Technologies, Montabaur, Germany.

For electrical characterization, aluminum contacts (~200 nm in thickness) with different areas were evaporated through a shadow mask on top of the hybrid dielectric film to yield metal–insulator–metal (MIM) structure composed of n-Si/Ta//dielectric hybrid film//Al (Figure 1). The intensity–voltage (I-V), capacitance–voltage (C-V), and capacitance–frequency (C-F) curves were measured in the mentioned MIM structure using an Agilent 4156 and HP 4277A Analyzers, at 1 MHz, from Agilent Technologies Inc. 395 Page Mill Road Palo Alto, CA 94303, USA.

Raman spectra were collected on the surface of the MIM ensemble presented in Figure 1 by using a LABRam HR800 spectrometer (Horiba France SAS, Palaiseau, France) equipped with a 325 nm laser (Kimmon Koha Co., Ltd., Tokyo, Japan) focused on films through an Olympus microscope objective of 40× NUV/0.47. The laser power was kept lower than 5 mW to prevent sample heating. ATR- FTIR spectra of the MIM ensembles were collected on a JASCO FT/IR-4700 spectrometer (Tokyo, Japan) using a Diamond Attenuated Total Reflectance (ATR) module and accumulating 64 scans at a resolution of 4 cm^−1^.

## 3. Results and Discussion

### 3.1. Thermal Analysis

**Figure 2:**
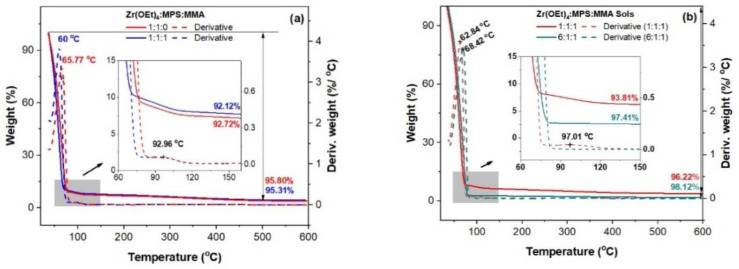
TGA-DTG of Zr(OEt)_4_:MPS:MMA as-prepared (**a**) and 24 h aged (**b**) sols.

The thermal decomposition of the as-prepared sols with different precursors ratios and corresponding aged sols, from ambient temperature up to 600 °C, shown in Figure 2, was investigated for the optimization of the treatment of the as-deposed gel thin films. The hybrid sol Zr(OEt)_4_:MPS:MMA (1:1:1) freshly prepared has a mass loss of 92.12% up to 150 °C, and loses another 3.2% up to 500 °C, where the mass stabilizes (Figure 2a). In the low-temperature domain (inset Figure 2a), three weight loss steps can be observed: a large and abrupt one (87%) between RT- ~70 °C assigned to the release of alcohols (parent solvent and precursors hydrolysis by-products), followed by a smaller gradual process (5%) with a maximum rate at ~93 °C, most probably corresponding to the release of water resulted by some precocious condensation of hydrolyzed precursors. The very small rate and weight loss over 120 °C seems the beginning of a complex process, with overlapping steps, of structuration of the gel resulted in the previous steps. By aging the 1:1:1 and 6:1:1 sols for 24 h, more visible differences can be observed between the two sol compositions (inset Figure 2b), namely the richest in zirconium ethoxide (6:1:1) sol underwent a bigger mass loss of 3.8%.

**Figure 3:**
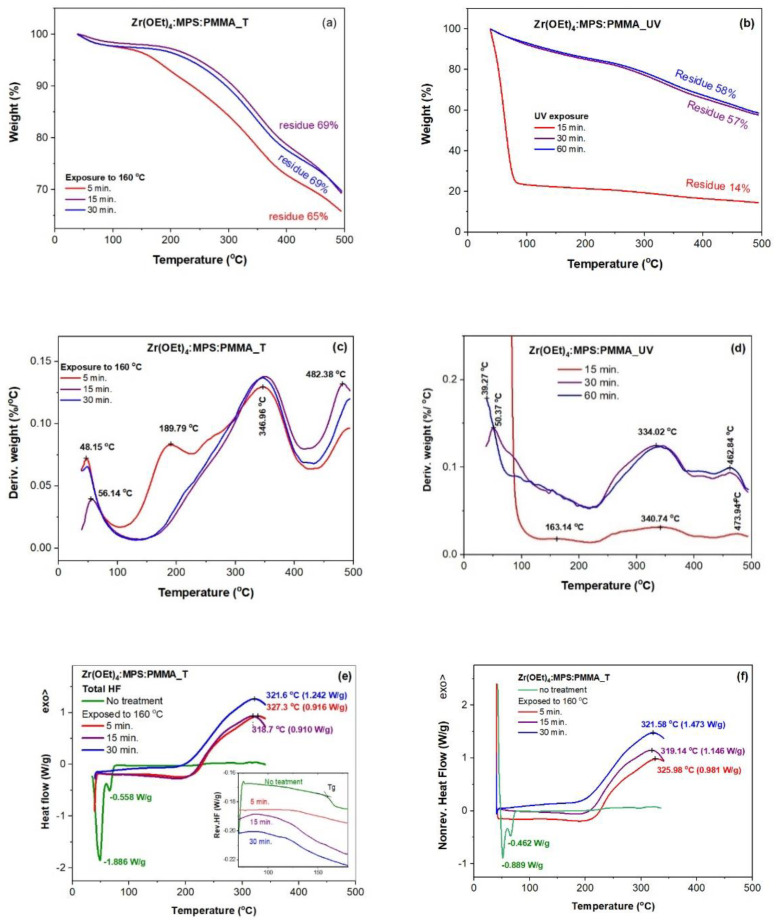
Thermal behavior, mTGA (**a**,**b**), mDTG (**c**,**d**), total HF (**e**), and non-reversing HF (**f**) of Zr(OEt)_4_: MPS: PMMA gel films cured at 160 °C, Zr(OEt)_4_:MPS:PMMA_T, or UV irradiated, Zr(OEt)_4_:MPS:PMMA_UV, for different time periods.

Figure 3 shows the TGA and DSC curves of ZrO_2_: MPS: PMMA hybrid gel films heat-treated (annealed) at 160 °C and/or UV irradiated for various spans. Annealed at 160 °C for 15 min. and UV exposed films for 30 min. trigged stabilization of the mass loss at 31% and 43%, respectively, between room temperature and 500 °C.

Two main decomposition ranges, e.g., RT−225 °C and 225–500 °C, are depicted in Figure 3a for the annealed gel films at 160 °C, over 5–30 min. range. The lowest temperature peak on the mDTG curves (Figure 3c) of the annealed samples at 48.15 °C and 56.14 °C corresponds to release of residual parent solvent and/or byproduct (ethanol and methanol) of precursors (Zr(OEt)_4_ and MPS) hydrolysis. Improved stability of the 15 min. annealed sample over the 30 min. and 5 min. annealed samples are depictable in Figure 3a. Intermediate character of the longest annealed sample might be caused by different distribution of the inorganic components. Kashiwagi et al. [26] reported three peaks in the derivative weight loss of the thermally degraded PMMA under nitrogen, e.g., 165, 270 and 360 °C for scissoring of the head-to-head linkages, vinylidene ends and randomly scission of the polymer backbone. The third type of scission prevails in the mDTG of the 5 min. annealed sample. Intriguingly, the shortest time annealed gel film has two main mDTG peaks (Figure 3c) within 100–430 °C range and a shoulder at about 255 °C analogous to the PMMA degradation under nitrogen. The position of the head-to head scission peak is almost 25 °C shifted towards higher temperatures very likely due to an in-situ restructuring process of the gel film taking place by hydrolyzed precursors condensation (exotherm), accompanied by elimination of condensation products (endotherm) [27], with compensation of thermal effects. This mechanism is confirmed by the vanishing of this peak from the mDTG curves (Figure 3c) and from the mHF curves (Figure 3e) for longer time annealed gel films (15 and 30 min). In addition, the most unstable H-H linkages in MMA oligomers were reported to give an DTG peak at about 195 °C [26]. Thermal behavior of the longer time annealed samples indicate that radicals obtained by scission of the H-H linkages are trapped and terminate by oxygen as expected for thermo-oxidative degradation of PMMA [26,27] where no DTG peak at 165 °C is observed. The only degradation process for longer annealed samples at about 347 °C corresponds to the random scission of the main chain of PMMA [26]. Since the weight loss mostly occurs up to 150 °C and the gel stabilizes within 15 min. (see mDTG curve in Figure 3c), the chosen annealing parameters of the hybrid sols were 160 °C and 10 min., to avoid (P)MMA degradation. Moreover, the glass transition temperature, T_g_, of the hybrid film without thermal and/or UV treatment is slightly <160 °C as indicated in the reversing heat flow curve of Figure 3e. 

UV photochemical activation of the hybrid materials has often reported in literature for faster processing/curing of denser films with lower content of impurities and no volatile emissions [16,28]. Hence, UV exposure was also applied for curing hybrid sols. The UV irradiated gel films for various spans show improved thermal stability as the exposure time increases (Figure 3b). Thus, the 15 min. irradiated film has low stability with 86% mass loss (Figure 3b), which is closer to that of the corresponding aged sol (Figure 2b). The 15 min. irradiated sample shows a mDTG peak at about 163 °C (Figure 3d) attributable to scission head-to head linkages as thermal degraded PMMA under nitrogen [26]. This also point out beginning of the gel structuring (see peaks at 163.14, 340.74, and 473.94 °C in Figure 3d). Prolongation of the UV exposure up to 30 and 60 min. causes a major modification of thermal behavior below 200 °C and disappearance of the 190 °C peak due to oxygen reaction with the radicals obtained by head-to head scissions in PMMA [26]. Thus, several stages of decomposition (~163, 334 and 462 °C) and elimination of volatile products at ~40 and 50 °C (Figure 3d) were recorded. These data indicate chemical structure change of the precursors before condensation processes, with formation of intermediates, which leads to several successive stages (depending on the UV exposure duration) of thermal degradation. Given the shape of the mDTG curve in Figure 3d, the post-deposition UV irradiation time of the films was chosen to be 30 min.

Hence, shorter time exposed gel films, e.g., 5 min. at 160 °C (Figure 3a,c) and 15 min. under UV irradiation (Figure 3b,d), encountered higher mass losses at low temperatures and smaller amount of the final residue due to weaker and/or incomplete interactions between components. Conversely, well-defined structure of the longer time stabilized gels, 15–30 min. at 160 °C and 30–60 min under UV irradiation, showed similar thermal behavior.

According to the DSC curves (Figure 3e,f), significant endothermic thermal processes below 100 °C for the untreated gel are no longer observed in the case of annealed samples for 15–30 min. at 160 °C. The latter ones have the exothermic effects above 200 °C, associated with significant mass losses (Figure 3a,c). Shape and temperature of the DSC peaks for the PMMA/ZrO_2_ hybrid films are highly influenced by the loading with zirconium building blocks of the PMMA matrix [29]. Thus, increased loadings cause increasing temperature of the DSC peaks as noticeable for the 15 min. annealed sample in Figure 3e.

The total heat flow curves (Figure 3e) and the nonreversible heat flow curves (Figure 3f) show maximum between 320–327 °C for all annealed gels. Subtraction of the non-reversing heat flow curve (kinetic information) from the total heat flow curve gave a maximum value for the reversing heat flow signal (heat capacity information) of 0.065 W/g for the 5 min. annealed sample and four times bigger values for the longer time annealed samples. This finding is in agreement with the TGA-DTG data showing that annealing at 160 °C for 15 min. and UV exposed films for 30 min. trigged stabilization of the mass loss at 31% and 45%, respectively between room temperature and 500 °C.

### 3.2. XPS Analysis

**Figure 4:**
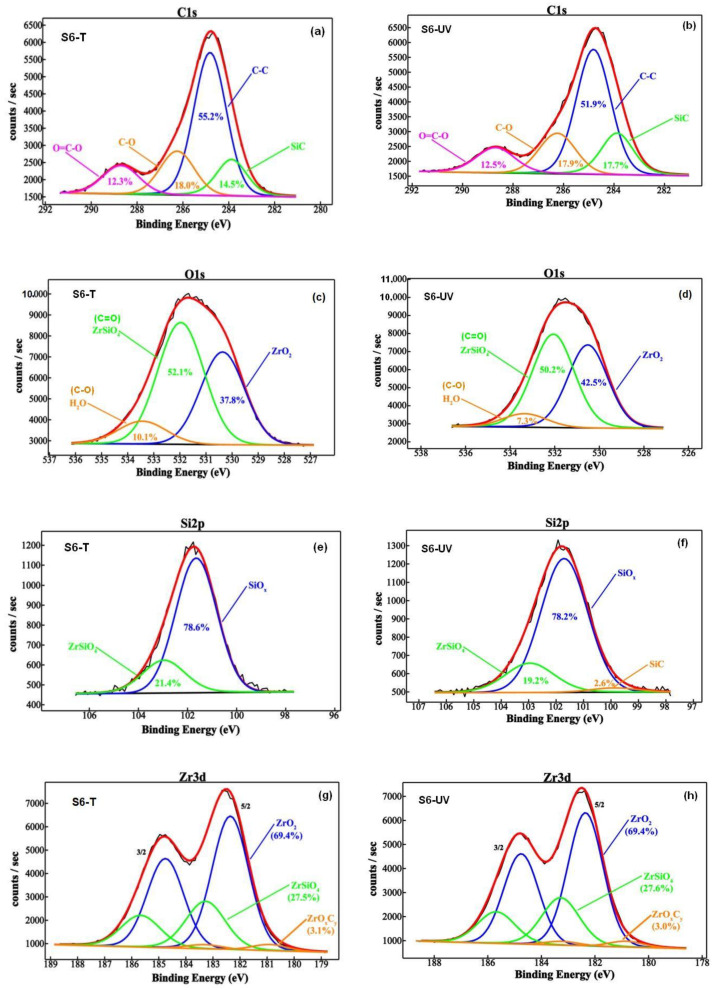
The deconvoluted spectra of the most prominent XPS transitions peaks, C1s (**a**,**b**), O1s (**c**,**d**), Si2p (**e**,**f**) and Zr3d (**g**,**h**), for films cured at 160 °C in air (S6-T) or UV irradiated (S6-UV).

In order to obtain surface information on both inorganic (zirconium and silicon chemical state) and organic components (carbon chemical state) and the corresponding elemental compositions, XPS analysis was performed (Figure S1). Deconvoluted curves of the most prominent transitions from XPS spectra of the zirconium-rich S6-(T/UV) samples, corresponding to C1s, O1s, Si2p, and Zr3d component elements states are illustrated in Figure 4. The difference lies in the amount of the chemical species corresponding to the component elements. Carbon presence on the surface of the S6-(T/UV) samples (Figure 4a,b) is alike with binding energies (BEs) of the C1s features assignable to the SiC, C-C, C-O, and O = C-O. Although oxygen signal was fitted with three components, additional contributions might be considered. The O1s sub-peak at 530.4 eV indicate higher integration of oxygens into the zirconium lattice (O^2−^) [30,31] for the UV-exposed material in comparison with the annealed sample. The main O1s sub-peak at about 531.9 eV was attributed to zircon ZrSiO_4_ [31] in Figure 4c,d is very closed to the one reported in literature (532.1 eV) for the double bonded oxygen in O-C = O from PMMA in PMMA-siloxane-silica coatings [32]. On the other hand, the third sub-peak can belong to oxygen in water as well as O-C = O [32]. This contribution is slightly smaller for UV-irradiated sample, S6-UV (Figure 4d).

Si2p spectra reveal the presence of silicon suboxides (SiOx, x < 2) from the peak position (2p = 101.6 eV) and ZrSiO_4_ with the 2p photoelectron line at 102.9 eV (see Figure 4e,f). By constraining the doublet area ratio and the spin orbit parameter, we can notice three deconvoluted contributions in the Zr3d spectrum (Figure 4g,h): the most intense feature at 182.3 eV is attributed to ZrO_2_ and the second component located at 183.2 eV can be assign to ZrSiO_4_. A tiny amount of ZrOxCy was detected at 180.8 eV (~3%). The lack of the C = C peak is noticeable, which means that not only in the presence of UV irradiation, but also the treatment at this low temperature of 160 °C, promoted the polymerization of MMA monomer. The quantitative data from XPS spectra shown in Figure 4 are summarized in Table S1. The elemental compositions in Table S1 reveal very similar atomic percentage in the investigated samples, with a very small increase for carbon and silicon and a small decrease for oxygen, and zirconium in the case of UV-treated sample, when comparing with the annealed one. Thus, treatment with UV irradiation increases the share of Si-C bonds (3.2%) and about the same (3.3%) decreases the relative concentration of C-C bonds. In conclusion, the share of the organic component (PMMA) decreases based on the formation of silicon carbide (2.6 at%, Table S1). The share of the hybrid phase with Zr-O-C bridges (ZrO_x_C_y_) only slightly decreases, from 3.1 to 3.0 (Table S1). The ZrO_2_/ZrSiO_4_ ratio seems to be unaffected (~2.5) by the type of the post-deposition treatment.

### 3.3. Vibrational Spectra

**Figure 5:**
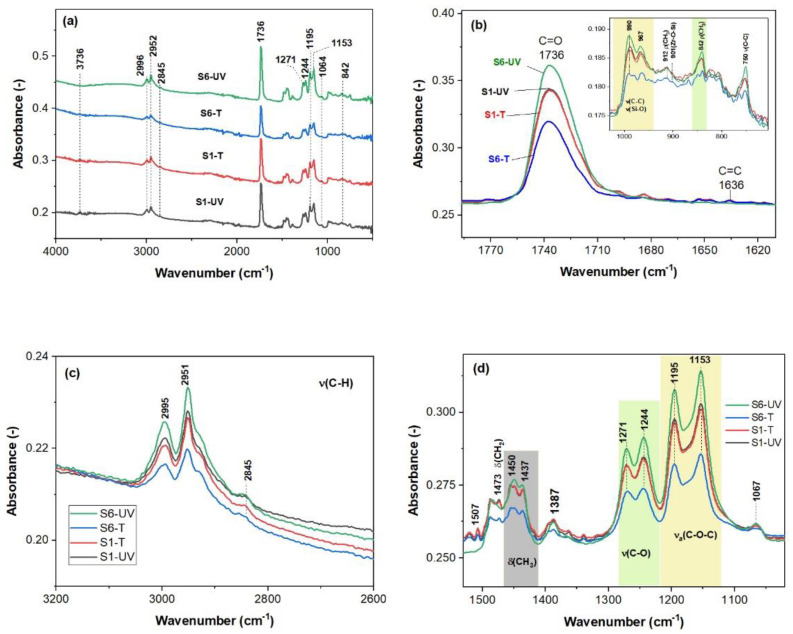
ATR-FTIR spectra of the cured films at 160 °C plate (S(1/6)-T) and UV irradiated (S(1/6)-UV) (**a**) and overlapped spectra within different regions (**b**–**d**).

ATR-FTIR spectral features in Figure 5 for the S1-(T/UV) samples are similar. All spectra illustrated in Figure 5a are dominated by the 1736 cm^−1^ band belonging to the C = O vibrations in PMMA, which is up-shifted in comparison with the MMA monomer and PMMA [1]. The annealed films at 160 °C show asymmetric carbonyl band (Figure 5b). 

Complete cure of the MMA monomer under UV light is noticeable for the S6-UV film due to the lack of the C = C vibrations at about 1636 cm^−1^ [15] in Figure 5b. This was also observed from the XPS date were no C = C bonds were depicted on the film surfaces. The tinny vinyl stretching band in the S(1/6)-T spectra may be caused by slight scissoring of the polymer chains within the films [33]. The high frequency bands at 2995 and 2951 cm^−1^ due to the C-H stretching vibrations of the CH_3_ and CH_2_ groups [23] are presented in all the spectra in Figure 5a. Minor peaks in the 3700–3800 cm^−1^ range due to Si-OH are present in all spectra except for S6-UV. The doublets at 1471/1244 and 1195/1153 cm^−1^ show presence of the C-O-C bonds in PMMA. Slightly modified spectral features within 1520–1320 cm^−1^ range, regarding absence of the δ(CH_2_) vibrations in terminal vinyl, were recorded for the S6-UV sample (Figure 5d). The 750 cm^−1^ band is also due to PMMA. Weak FTIR vibrations of the Zr-O and Si-O bonds are present within 850–500 and 1100–1050 cm^−1^ regions [34]. Moreover, weak shoulder at about 800 cm^−1^ of the broad band over 800–1100 cm^−1^ (inset Figure 5b) belongs to Zr-O-Si bonds [34].

**Figure 6:**
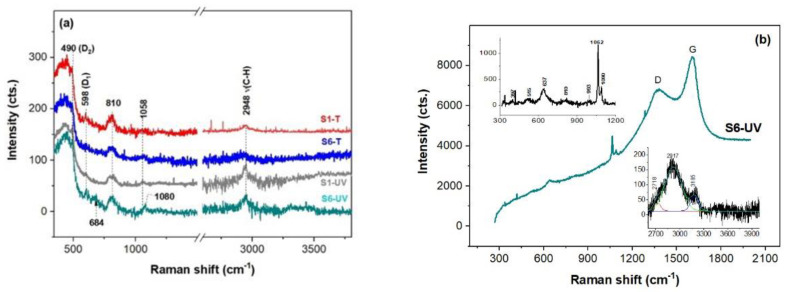
UV-Raman spectra of the thin films (**a**) cured at 160 °C plate (S1-T, S6-T) and UV irradiation (S1-UV, S6-UV) and (**b**) collected near metallic contacts.

UV-Raman spectroscopy (325 nm) allows fluorescence removing and recording spectra of thin films of nanomaterials due to increased sensitivity as a consequence of resonance effect. Thus, all the investigated PMMA/ZrO_2_ films show peaks at 460, 597, ~600, ~800, and 1058 cm^−1^ (see Figure 6a) assignable to m-membered cyclosiloxane rings (m ranges from seven to three), symmetrical Si-O-Si stretch and transverse-optical Si-O stretch [35]) very likely due to silanization with MPS [36] of the inorganic component, e.g., zirconium-based compounds as pointed out by XPS data. Vibration modes of PMMA at 604, 818 cm^−1^ attributable to symmetric stretching modes of C-C-O and C-O-C ester [37] overlap with silica modes. However, the C-H stretching vibrations of the O-CH_3_, α-CH_3_ and asymmetric stretch of the CH_2_ groups in PMMA [38] are present in Figure 6a for all the films investigated here. No peak at 660 cm^−1^ assignable to ZrC [39] is depicted for the S(1/6)-T films unlike the small contribution at about 680 cm^−1^ for the UV exposed films.

Distinct Raman spectra were recorded for S6-UV film in the proximity of the aluminum contacts (Figure 6b). Thus, two wide and intense bands at about 1375 cm^−1^ (D band due to breathing modes of sp^2^ carbon atoms in rings) and 1605 cm^−1^ (G band attributable to bond stretching of all pairs of sp^3^ atoms in chains and rings [40]) of the S6-UV spectrum originate from amorphous carbon. Regardless content of the sp^2^ hybridized carbon atoms its Raman bands are very intense. Since the sp^2^ hybridized carbon materials are strong scatterers, their D and G bands are very intense. The 2D region at high frequency is peaking up at 2917 cm^−1^ (inset Figure 6b). Sharp peaks within 1055–1071 cm^−1^ were also reported for C(sp^3^)-C(sp^3^) bonding since UV-Raman spectroscopy is also sensitive to the carbons with sp^3^ hybridization [41,42]. The 637 cm^−1^ peak might belong to monoclinic and tetragonal zirconia (m- and t-ZrO_2_) [43].

### 3.4. SEM, TEM and AFM Analysis

**Figure 7:**
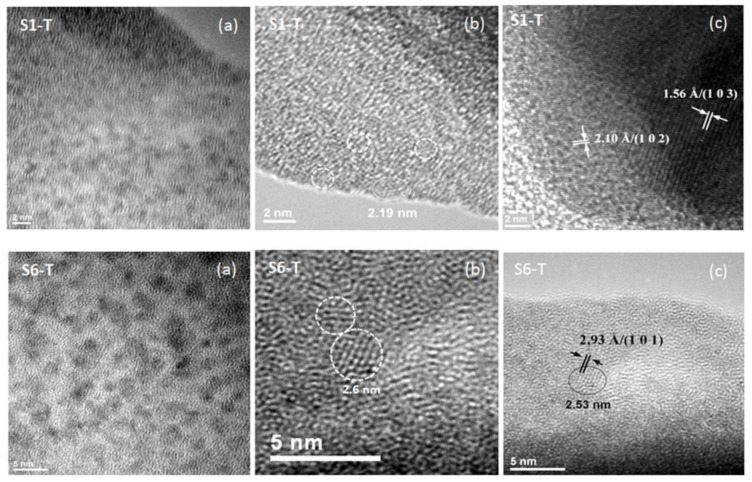
TEM images (**a**), high resolution TEM (HR-TEM) images (**b**) and the orientation of atomic plane in the crystal lattice (Miller indices) (**c**) of the hybrid films.

The surface and cross-section SEM images show homogeneous one-layer thin films morphology, with varying thickness, depending on the ratio between the components and the post-deposition treatment, between 61–75 nm (Figure S2 and Table S2). The light field transmission images confirmed homogeneously distributed nanoparticles inside the hybrid films (Figure 7a). Slightly larger ZrO_2_ crystallites of around 2.5 nm in diameter with the orientation of the Miller indices (101) and interplanar distance of 2.93 Å were observed in the film with increased (6:1) Zr(OEt)_4_:MMA molar ratio (S6-T) identified as tetragonal ZrO_2_ (Figure 7b). By measuring the interplanetary distances in the high-resolution images (Figure 7b,c) were identified the orientation with the Miller indices (102) and (103) and interplanar distance of 2.10 Å and 1.56 Å, respectively, corresponding to the crystallographic planes of tetragonal ZrO_2_ (ICDD 01-078-3194) in the sample with 1:1:1 ZrO_2_: MPS: PMMA molar ratio (S1-T).

The X-ray patterns of samples cured at 160 °C are presented in Figure S3. Crystalline phases corresponding to tetragonal ZrO_2_ (ASTM 00-070-7303) were identified from the XRD patterns, confirming the results of HR-TEM (Figure 7), and cubic Ta (ASTM 04-014-0145). This is also in agreement with the Raman information on zirconia polymorphs. The values of the average crystallite diameter of ZrO_2_ phase, calculated with Scherer’s formula, are 2.32 and 2.87 nm, for S1-T and S6-T samples, respectively. As mentioned above, the characteristic diffraction peaks of tantalum come from the metallic layer on which the hybrid film was deposited.

**Figure 8:**
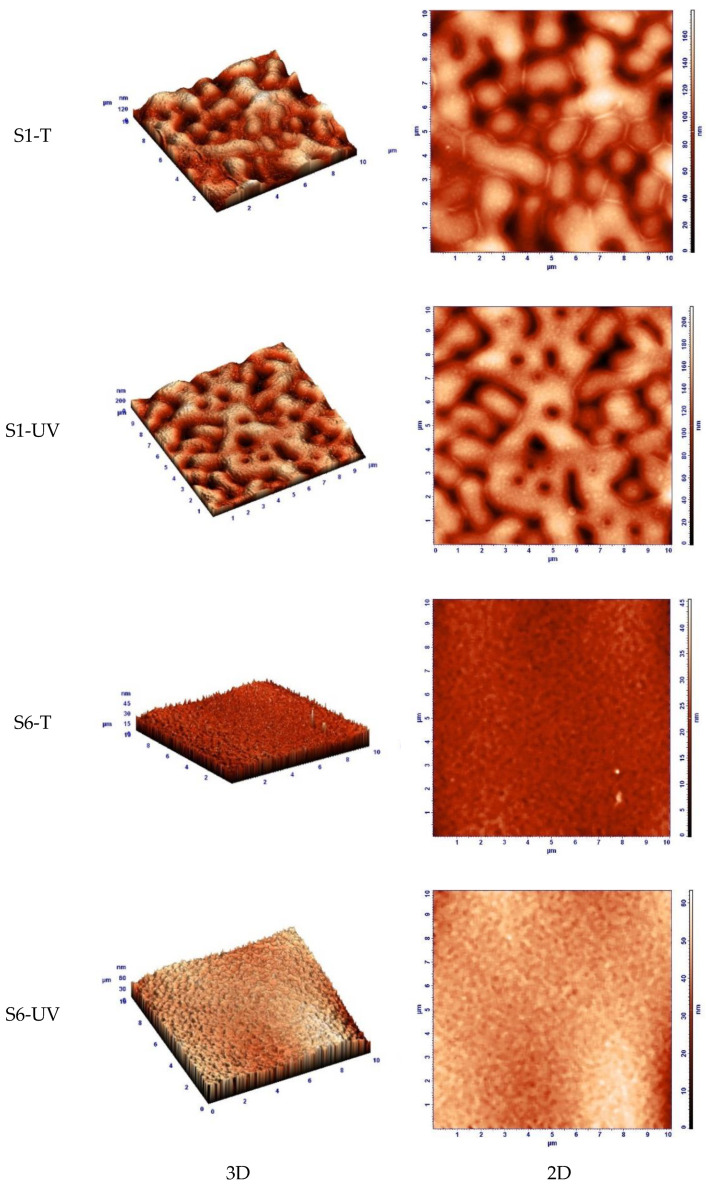
2-D and 3-D AFM images of the cured hybrid films.

More detailed nanoscale morphology of the obtained films was observed by means of AFM investigation. The 3D and 2D and phase composition AFM images in Figure 8 show that varying the component ratio significantly change the film morphology. In addition, post-deposition annealing treatment affects the morphology. For films with the same composition, the post-deposition heat treatment at 160 °C produces shrinkage of the structure, compared to UV irradiation with less compact structure as depictable from film thickness in the corresponding SEM images in Figure S2. Otsuka and Chujo [34] reported that surface roughness increases as amount of ZrO_2_ nanocrystals increases. Surface roughness of the S(1/6)-(T/UV) films is a subject of zirconium content but also post-deposition treatment used (Figure S4). Analyzing the phase contrast images in Figure S4 (left column) a maximum within 1 ÷ 4° range due to a surface morphology without phase separations is observed. Therefore, considering this coefficient range these films are very uniform, regardless of how S(1/6)-(UV/T) films are obtained and treated.

Analysis of the Root Mean Square (RMS) as a function of the side square of the investigated area (L) pointed out that higher values for the S1-(T/UV) samples (in the range 25–35 nm) than the S6-(T/UV) samples with values of 5–15 nm. Moreover, the RMS values change slightly as the area under investigation increases, meaning that each film is very uniform, with no randomly distributed defects. In addition, according to the single Gaussian curve of the histograms illustrated in Figure S4a unique phase is present in all investigated samples. The narrower Gaussian peak, the better dimension uniformity of the nanometric surface unevenness is recorded.

Hence, ZrO_2_ phase is more homogeneously distributed in the rich-zirconium hybrid PMMA-based matrix, i.e., the S6 samples. These observations are very important in obtaining thin films for opto-electronics. A crucial factor influencing distribution of the inorganic nanocrystals into the organic matrix is the covalent bonding between PMMA and ZrO_2_ by means of the coupling agent [34]. In the present case, post-deposition treatment for curing the organic matrix also influence quality of the hybrid films.

### 3.5. Mechanism of Chemical-Structural Transformations

Basically, our synthesis approach is a modified alkoxide-based sol-gel method, the most suitable for production of highly homogeneous hybrid organic-inorganic complex materials [7]. The classical sol-gel route was modified by adding a vinyl-silane type (MPS) network former precursor to the zirconium ethoxide. Finally, the MMA monomer organic connector was added to control the structural and morphological homogeneity of the final product [7,44]. Three main chemical processes can be considered in formation of hybrid nanostructured NBB-based polymeric network of the dielectric films presented in this work:

*I*.  *Hydrolysis of precursors*

Tendency towards hydrolysis and polymerization of the alkoxides is based on the metal attached oxygens with a bigger electron density. These oxygen atoms can be better donors than the ones from organic donor molecules as alcohols and ethers [45]. By hydrolysis of the alkoxy groups of Zr(OEt)_4_ and MPS, hydroxyl–metal species are formed during sol preparation (Reactions (1) and (2)):


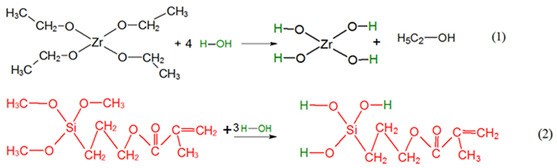

*II*.  *Formation of nBBs*

During the sol ageing, in situ formation of hybrid zirconia-siloxane nBBs occurs by polycondensation of hydrolyzed precursors with formation of Zr-O-Si oxo bridges. Depending on the Zr(OEt)_4_: MPS ratio, this reaction competes with the formation of homogeneous Zr-O-Zr clusters, representing the core of the functionalized metal–oxo cluster representing the hybrid structural units (nBB) (Reaction (3)).


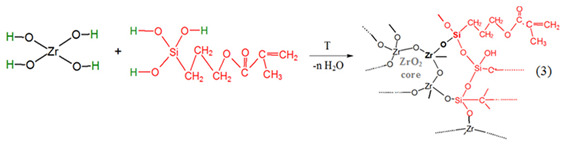


Structure of the resulted hybrid nBB depends not only on relative molar ratio of the two precursors, but also on relative contribution of the Reactions (1) and (2), i.e., their hydrolyzation rates [24,45].

*III*.  *Formation of hybrid network of thin film by nBBs—MMA polymerization*

In contrast with classical sol-gel method where material framework is formed by successive hydrolysis (1) and polycondensation Reactions (2), final structure of the hybrid film obtained by a modified sol-gel method is determined by organic linker interaction with the previously formed framework. This study is about copolymerization reaction between C = C terminal group of the organosilane functionalized oxo cluster [-Zr-O-Si-….C = C], formed in the previous step, with vinyl group of the MMA monomer.

The Reaction (4) illustrates covalent addition of the MMA organic linker to the core–shell zirconia-silane nBB-based structural units by co-polymerization, with formation of an outer MMA layer. This reaction can compete with formation of MMA oligomers by MMA-MMA polymerization.


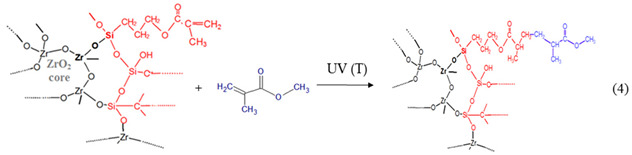


During annealing or UV irradiation, the methyl acrylate groups (COO-CH_3_) of the MMA or MMA and PMMA outer layer promotes the end-group mediated supramolecular self-assembly [46] of the resulted MMA-grafted silane-functionalized zirconia nBBs. SEM (Figure S2) and especially AFM (Figure 8 and Figure S4) showed different morphology of the investigated films. Homogeneous morphology of the rich-zirconium hybrid films (S6 samples) is consistent with formation of the self-assembled nBBs-based supramolecular structure containing homogeneous dispersed zirconia-MPs core–shell units into organic matrix [47,48].

Conversely, the 2D and 3D AFM images (Figure 8) illustrate different surface morphology for poor-zirconium hybrid films (S1 samples). It is known that the structure of the copolymer films is mainly determined by the molar ratio between the two monomers and by the post-deposition treatment of the wet gel film [6]. In this paper, two groups of thin films with (Zr:Si):MMA molar ratio of (1:1):1 and (6:1):1 were investigated. In the case of films with of 1:1:1 molar ratio composition, there is the highest probability of achieving a maximum number of Zr-O-Si-C hybrid bridges, given that Zr(OEt)_4_ contains four hydrolysable groups and the silicon of MPS only three ones, leading to Zr(OH)_4_ and R-Si(OH)_3_ respectively products, assuming complete hydrolyzation reactions. An anisotropic growth in the S1-(T/UV) samples, where each Zr atom forms Zr-O-Si bridges (Reaction (5)), can explain the AFM images in Figure 8.


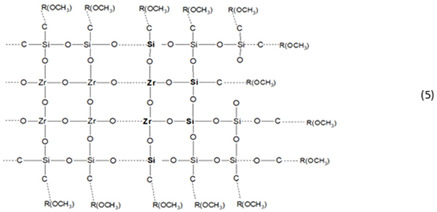


The outer MMA or MMA and PMMA layer ensures cohesion between these structural units by self-assembling [46] into a worm-like structured film [48], as can be seen from the AFM images (Figure 8). As mentioned before, the AFM phase contrast analysis (Figure S4) confirmed no phase separation structure over 1 ÷ 4° range for both compositions of the investigated films.

### 3.6. Optical and Electrical Properties Behavior of Thin Films

**Figure 9:**
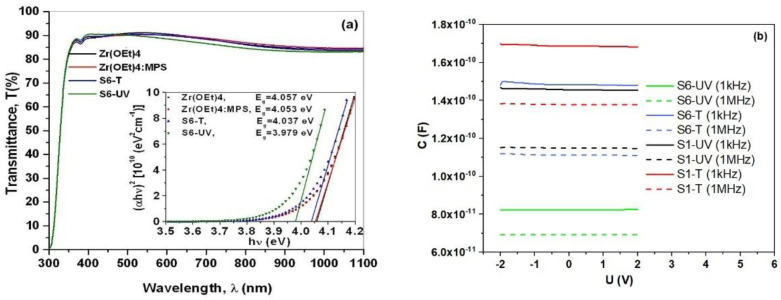
Optical spectra (**a**) and C-V characteristics (**b**) of cured hybrid films. Zr(OEt)_4_ precursor and MPS modified Zr(OEt)_4_ were used for comparison.

Homogeneity of the ZrO_2_-MPS-PMMA hybrid films was also investigated by UV-VIS transmittance in comparison with the pristine and MPS-modified Zr(OEt)4 (Figure 9a). Lower transmittance in the visible light for the S6-UV sample in comparison with the annealed counterpart of S6-T is depictable in Figure 9a. A plausible explanation of this behavior might consist in forming aggregates of cross-linked nanocrystals. Almost similar transparency was recorded for hybrid sample of S6-T and the PMMA free samples of Zr(OEt)_4_ and MPS- Zr(OEt)_4_.

The hybrid films exhibit dielectric behavior, with a very good symmetry and linearity in the interval [−2 V ... 2 V] (Figure 9b). The relative permittivity reduces with frequency (from 1 kHz to 1 MHz), which is in line with theory of dielectric materials [47]. At higher frequencies the more inert dipoles cannot follow the exciting signal, so capacity reduces with increasing frequencies. Furthermore, the decay is roughly identical in the order of 20% for all of the samples suggesting that the nature of the dipoles is more or less independent on concentration or annealing scheme. Yet, the density of dipoles active in that frequency range is about 40% to 70% higher in solely hot plate annealed samples in respect to the photonically (UV) annealed ones. Extracted values for the dielectric function in Table S2 vary between 17.9 at 1 KHz (14.6 at 1 MHz in Table S3) for S1-T and 7.9 at 1 KHz (6.35 at 1 MHz in Table S3) for S6-UV, exceeding values of PMMA (3.2 at 1 MHz) or insulators as SiO_2_ (3.9 at 1 MHz) [44]. Dielectric permittivity of a film is proportional to its electronic polarization [44,45].

When studying the I-V curves (Figure S5), except S6-UV film that has symmetrical exponential characteristics for positive and negative voltage, without hysteresis. The other films thin films do not behave uniformly. They exhibit a strong hysteresis, for negative applied voltage, while the corresponding phenomenon for positive applied voltage is weaker. This is unlike the usual symmetrical hysteresis, such as for ferroelectric materials, but it can be found in cases of hybrid materials with crystalline/amorphous phases films, leading to a polarity dependence in the leakage currents [49,50]. The branches of the negative hysteresis connect around −3 V and the coercive voltage is around −2 V. According to the curve shape, it is assumed that two different conduction mechanisms or respective defect types contribute to the conduction characteristics. The shape of the I-V characteristics in negative direction is intermediate, between exponential (similar to the curve observed for the UV-annealed sample) at low voltages and linear below −3 V. The thermally annealed sample with lower concentration of Zr(Eth)_4_ shows similar behavior albeit lower current levels and slightly stronger hysteresis for both polarities. In terms of resistivity, the conducted current spans from 10^−9^ A at 3 V for S6-UV to 10^−8^ A at 3 V for S6-T. When considering the chosen film thickness and area the average resistivity (at a voltage of 3 V) spans from 12 × 10^+8^ Ωm to 12 × 10^+9^ Ωm, which means a good insulation behavior.

Samples with richer inorganic phase show lower capacity than those with low concentration. In this case, either more Zr is bound into ZrO_2_ particles, reducing the respective defects generated by possible precursor residues or, more likely, the reduced total particle surface of ZrO_2_ particles (which are ~2 times in volume with respect to low-Zr(OEt)_4_ samples) better matches the available crosslinker and MMA concentrations to reduce the total dipole density in the organic regime.

## 4. Conclusions

In this work we report a modified sol-gel method by adding a vinyl-silane type (MPS) network former precursor to the zirconium ethoxide and MMA monomer organic connector to control the structural and morphological homogeneity of the hybrid nanostructured NBB-based polymeric network dielectric films. Except for simplicity, this method does not use toxic and/irritant solvents. The obtained thin films of ZrO_2_-MPS-PMMA have leakages within 10^−12^ A and 10^−7^ A for applied voltage in the ±(1–2) V and ±6 V ranges, respectively for a film thickness of 61–75 nm. Varying the (Zr:Si): MMA molar ratio from (6:1):1 to (1:1):1, structure of the hybrid films changes from core–shell NBB-based supramolecular self-assembled units to worm-like structures. 

Tuning of the dielectric properties is achievable by selection of the optimum precursors ratio of the three precursors used (Zr(OEt)_4_-MPS-MMA), post-deposition curing treatment (heat treatment and/or UV irradiation) as well as its duration. No phase separation was depicted from the AFM finding of the films under investigation. Furthermore, the rich-Zr(OEt)_4_ samples also have smoother morphology of the film surface due to optimized mixing of the components. Enhanced crosslinking between the organic matrix and the inorganic building blocks of the UV cured samples was supported by XPS and Raman findings as well as C-V and I-V characteristics pointing out a lower amount of the residual dipoles. Consequently, characteristics of the 6:1:1 mixture prevail over the ones of the 1:1:1 mixture, while UV curing caused formation of less residual defects and better stability than thermal annealing. The resulting k value of around 17 strongly exceeds the base value of PMMA and even classical insulators like SiO_2_ indicating an excellent applicability in electron devices. Maintaining high optical transparency better than 85% in accordance with the film thickness and flexibility show that these films are good candidates for transparent and flexible electronics.

## Data Availability

The data presented in this study are contained within the article.

## Supplementary Materials

The following supporting information can be downloaded at: https://www.mdpi.com/article/10.3390/gels8020068/s1, Figure S1: The superimposed XPS survey spectra for the S6-T and S6-UV samples; Figure S2: SEM images of the investigated samples; Figure S3: XRD patterns of the S(1/6)-T samples cured at 160 °C; Figure S4: Phase contrast images and histograms of the cured films; Figure S5: I-V Curves of the S1-T and S6-T samples; Table S1: Elemental composition and calculated relative concentrations (at.%); Table S2: Dielectric constant of thin films from capacitance measurements at 1 kHz; Table S3: Dielectric constant of thin films from capacitance measurements at 1000 kHz.
