# Supplementary material for: Low-Temperature and UV Irradiation Effect on Transformation of Zirconia -MPS nBBs-Based Gels into Hybrid Transparent Dielectric Thin Films
Source: Gels. 2022 Jan 20;8(2):68. doi: 10.3390/gels8020068 (PMC8870923; doi:10.3390/gels8020068)
Supplement: Supplementary file 1 [file gels-08-00068-s001.zip › gels-1534600-supplementary.pdf]

SUPPLEMENTARY INFORMATION

Low-Temperature and UV irradiation effect on the transformation of zirconia -MPS NBBs-based gels into hybrid transparent dielectric thin films

Viorica Muşat[1*], Elena Emanuela Herbei[1], Elena Maria Anghel[2*], Michael P.M. Jank[3], Susanne Oertel[3], Daniel Timpu[4] and Laurenţiu Frangu[5]

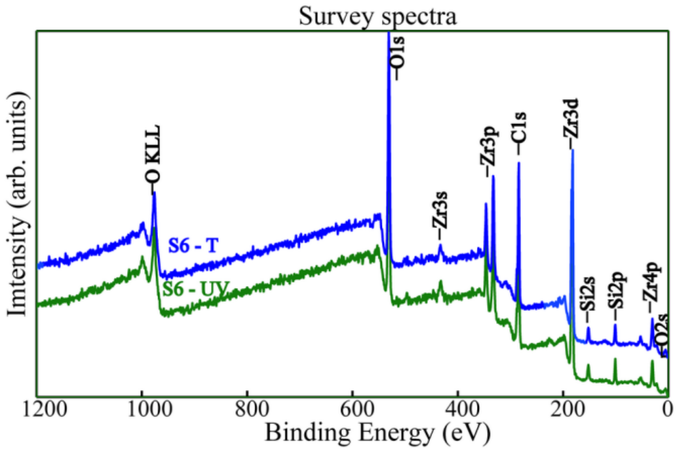

Figure S1. The superimposed XPS survey spectra for the S6-T and S6-UV samples.

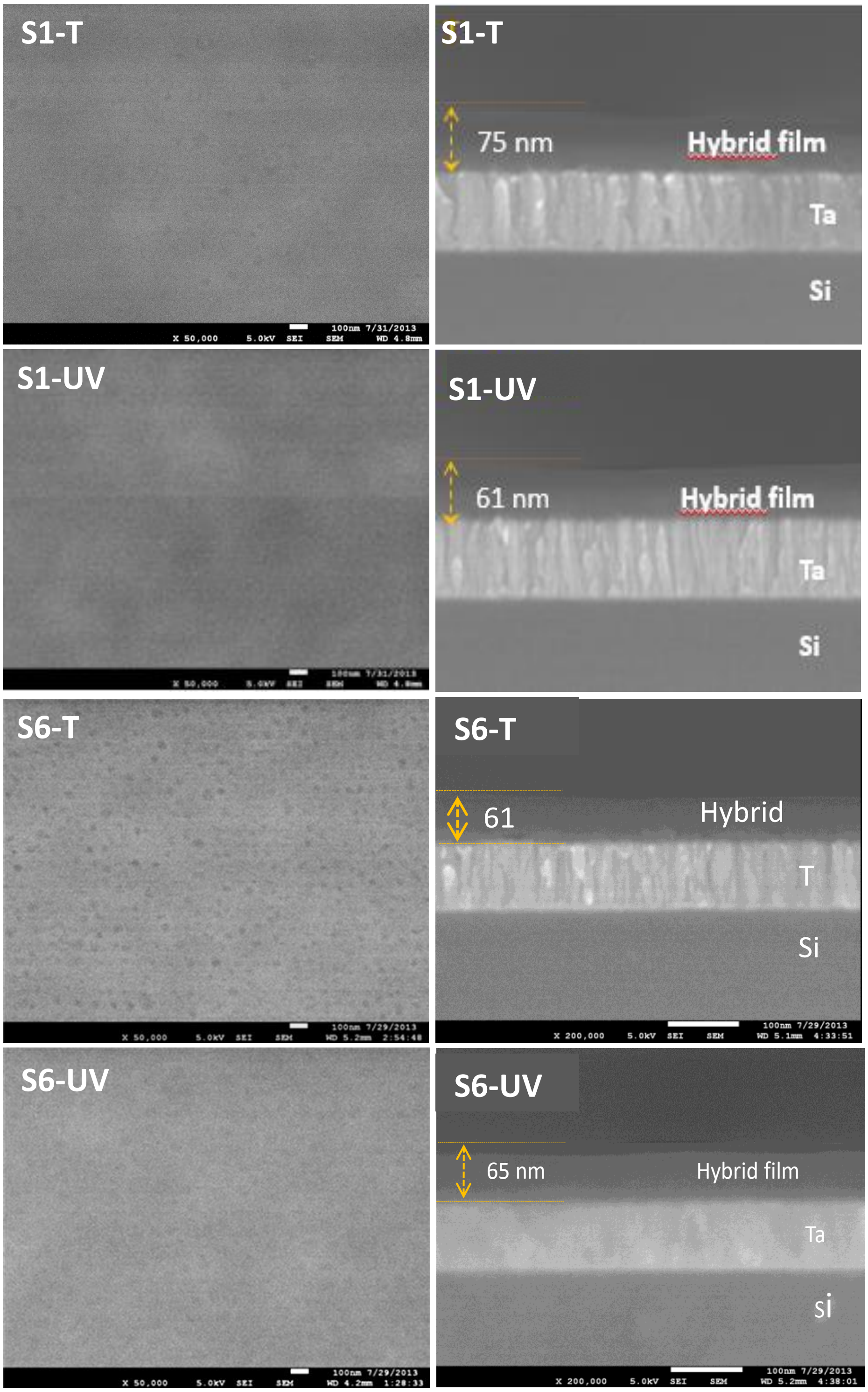

Figure S2. SEM images of the investigated samples.

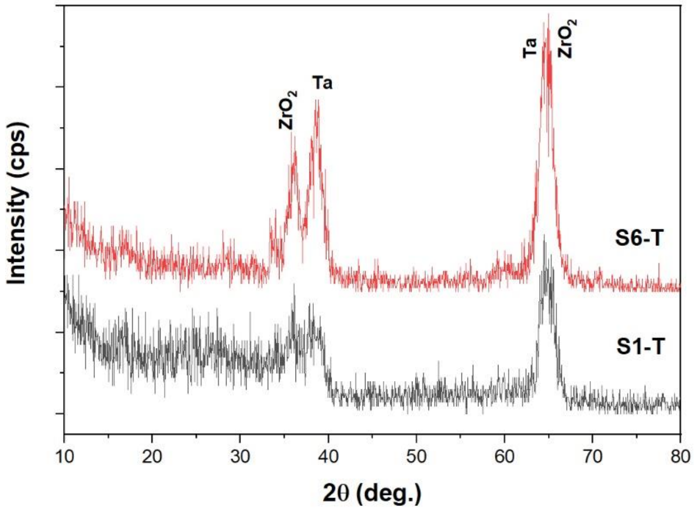

Figure S3. XRD patterns of the S(1/6)-T samples cured at 160°C.

S1-T	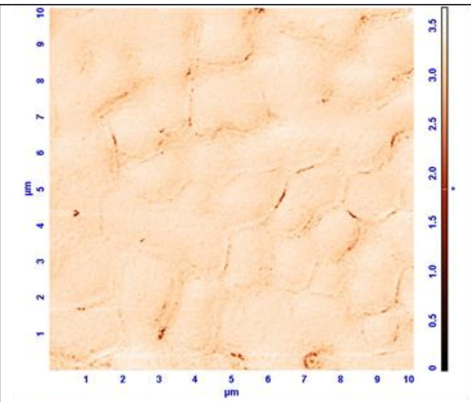	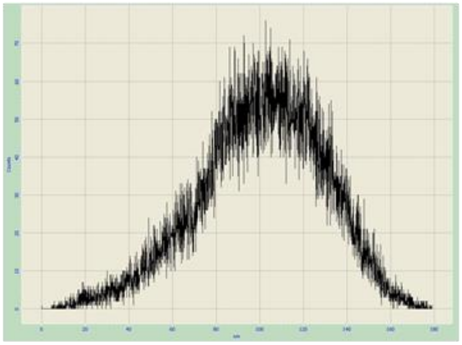
S1-UV	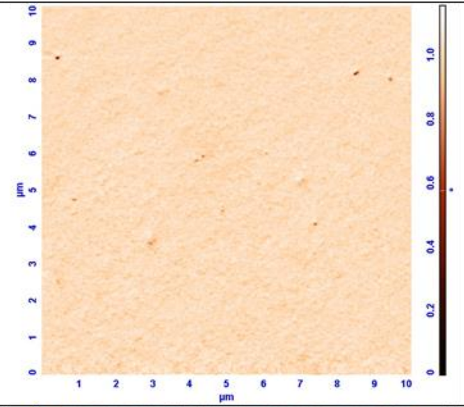	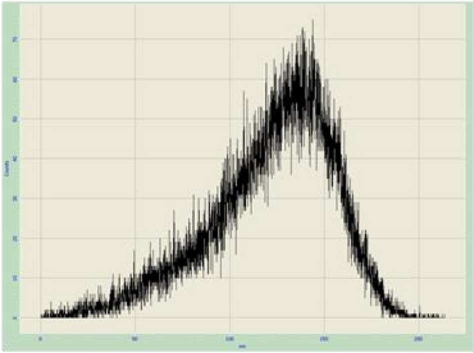
S6-T	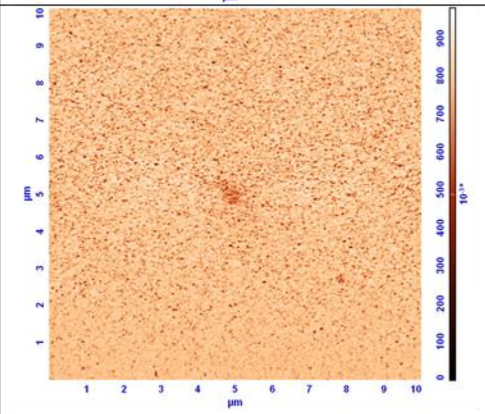	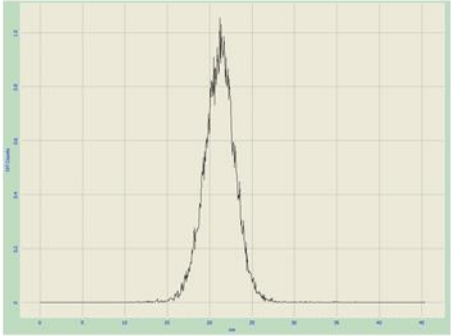
S6-UV	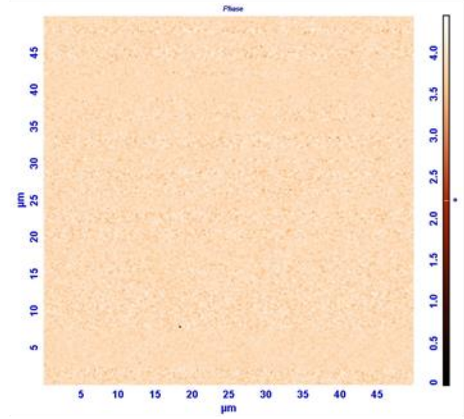	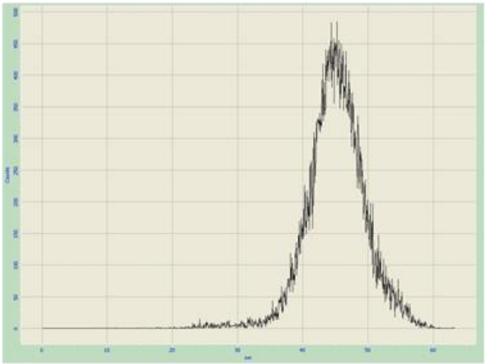
	Phase contrast	Histograms

Figure S4. Phase contrast images and histograms of the cured films.

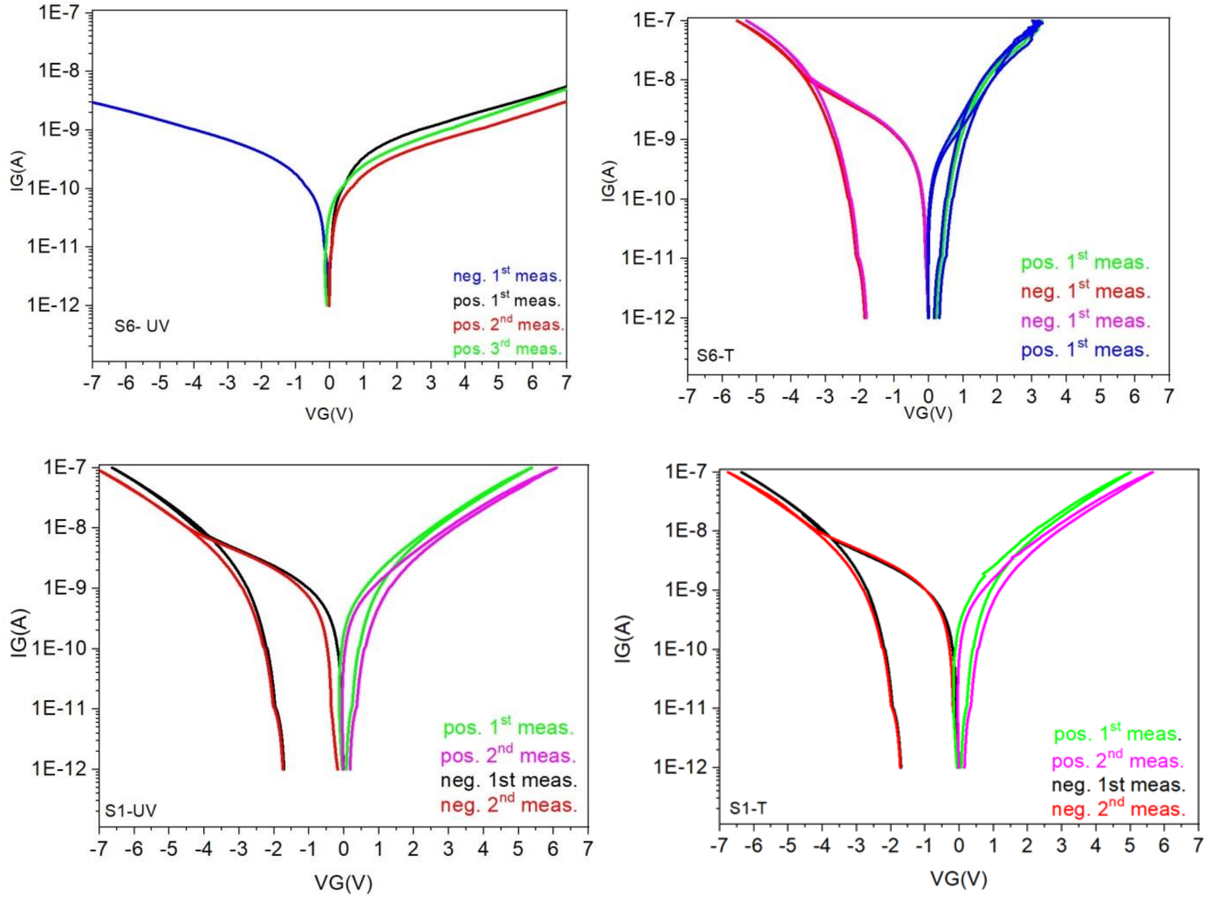

Figure S5. I-V Curves of the S1-T and S6-T samples.

.

Table S1. Elemental composition and calculated relative concentrations (at.%).

Sample	Elemental composition (at%)				Calculated relative concentrations				
	**C1s**	**O1s**	**Si2p**	**Zr3d**	**Si-C/** C1s	**C-C/** C1s	**SiC/** Si2p	**ZrO_2** Zr3d	**ZrO_2/ $ZrSiO_4$**
**S6-T**	49.2	37.8	4.9	8.1	14.5	55.2	-	69.4	2.52
**S6-UV**	49.7	37.4	5.2	7.7	17.7	51.9	2.6	69.4	2.51

Table S2. Dielectric constant of thin films from capacitance measurements at 1 kHz.

Sample	Electrode area/surface (m^2)	Film Thickness d (m)·10^{-9}	Capacitance C (F)	Dielectric constant (k)
**S1-UV**		61	$1.468\cdot10^{-10}$	12.7
**S1-T**	$8\cdot10^{-8}$	75	$1.698\cdot10^{-10}$	17.9
**S6-UV**		65	$8.249\cdot10^{-11}$	7.6
**S6-T**		61	$1.482\cdot10^{-10}$	12.7

Table S3. Dielectric constant of thin films from capacitance measurements at 1000 kHz.

Sample	Electrode area/surface (m^2)	Film Thickness d (m)·10^{-9}	Capacitance C (F)	Dielectric constant (k)
**S1-UV**		61	$1.152\cdot10^{-10}$	9.92
**S1-T**	$8\cdot10^{-8}$	75	$1.383\cdot10^{-10}$	14.6
**S6-UV**		65	$6.919\cdot10^{-11}$	6.35
**S6-T**		61	$1.119\cdot10^{-10}$	9.64
